# INTEGRATING SOCIAL NEEDS CARE INTO THE DELIVERY OF HEALTH CARE TO IMPROVE THE NATION’S HEALTH FOR OLDER ADULTS

**DOI:** 10.1093/geroni/igz038.1840

**Published:** 2019-11-08

**Authors:** Tamara J Cadet, Robyn Golden, Keegan Warren-Clem

**Affiliations:** 1 Simmons University, Boston, Massachusetts, United States; 2 Rush University Medical Center, Chicago, Illinois, United States; 3 The University of Texas at Austin School of Law and McCombs School of Business, Austin, Texas, United States

## Abstract

An ad hoc committee of the National Academies of Sciences, Engineering, and Medicine examined the potential for integrating services addressing social needs and the social determinants of health into the delivery of health care to achieve better health outcomes and to address major challenges facing the U.S. health care system. These challenges, include persisting disparities in health outcomes among vulnerable subpopulations, often defined by a number of factors including age. Presenters will discuss and provide recommendations in the following areas: 1. evidence of impact of social needs care on patient and caregiver/family health and wellbeing, patient activation, health care utilization, cost savings, and patient and provider satisfaction; 2. opportunities and barriers to expanding historical roles and leadership of social workers in providing health-related social needs care and evidence-based care models that incorporate social workers and/or other social needs care providers in interprofessional care teams across the care continuum (e.g., acute, ambulatory, community-based, long-term care, hospice care, public health, care planning) and in delivery system reform efforts (e.g., enhancing prevention and functional status, care management, and transitional care; improving end-of-life care; integration of behavioral, mental, and physical health services); and 3. realized and potential contributions of social needs care to make health care delivery systems more community based, person- and family/caregiver-centered, and responsive to social and structural determinants of health, particularly for vulnerable populations and communities, such as older adults and low-income families. Examples for each of the three areas will also be presented.

